# Clinical Radiologic Analysis of 2964 Papillary Breast Lesions

**DOI:** 10.1155/tbj/5639017

**Published:** 2024-12-24

**Authors:** Hanyu Zhang, Anbang Hu, Yanling Li, Mingcui Li, Zhiyuan Rong, Weilun Cheng, Yansong Liu, Yunqiang Duan, Jianyuan Feng, Ziang Chen, Tianshui Yu, Jiarui Zhang, Ting Wang, Yuhang Shang, Zhengbo Fang, Jiangwei Liu, Fanjing Kong, Fei Ma, Baoliang Guo

**Affiliations:** Department of General Surgery, Second Affiliated Hospital of Harbin Medical University, 246 Xuefu Street, Nangang District, Harbin, China

**Keywords:** breast cancer, papillary breast lesions, risk factors, treatment

## Abstract

**Background:** Clinical management of papillary breast lesions (PBLs) remains controversial. Our objective was to analyze the independent risk factors associated with malignant PBLs.

**Methods:** A retrospective review of clinical variables in 2964 patients with papillary lesions available for evaluation.

**Results:** This retrospective study screened that older patients (≥ 50 years), postmenopausal, palpable tumor, tumor size ≥ 15 mm, peripheral tumor, and tumor with calcification were independent risk factors affecting malignant PBLs. Moreover, the probability of malignant PBLs without the appealed risk factors was only 3.4%.

**Conclusions:** Patients without risk factors for papillary lesions can manage their health with imaging surveillance. The choice of surgical treatment for the remaining patients is a reasonable recommendation.

## 1. Introduction

Papillary breast lesions (PBLs) are common proliferative disorders of the breast, which present mostly with arborizing fibrovascular stroma as core of the papillae. Each papilla is covered by epithelial cells, with or without a myoepithelial cell layer. PBLs present as a wide range of lesions including papilloma, papilloma with atypical ductal hyperplasia/DCIS, papillary DCIS, and solid and encapsulated papillary carcinomas in situ. Among them, intraductal papillomas (with or without atypia) were found in 5.3% of benign breast biopsies from a cohort of > 9000 women [1]. PBLs can occur in women of all ages, usually in their 30s and 50s, clinically appear as mass lesions and/or nipple discharge [2]. There are few specific clinical features differentiating between benign and malignant papillary lesions [3, 4]. Immunohistochemical stains provide diagnosis indicator by CK5, ER, p63 expression, and etc. [5]. Pure intraductal PBLs have a low upgrade rate (1%–9%), whereas lesions with concomitant atypia have been reported to have a rate of up to 38%. However, the morphogenesis of PBLs is not well understood. European Third International Consensus Conference discussed about the examination and diagnosis of PBLs as B3 lesions, including core needle biopsy (CNB), vacuum-assisted biopsy (VAB), and open excision (OE) [6]. However, the balance between biopsies and pathology decision has been brought up as an unsolved question [7–10]. Thus, in this study, we explore on the data of 2964 patients with papillary lesions aiming to form a retrospective study of independent risk factors associated with malignant PBLs through clinical variables as an adding analysis for early PBLs diagnosis.

## 2. Materials and Methods

### 2.1. Patients

**Figure 1:**
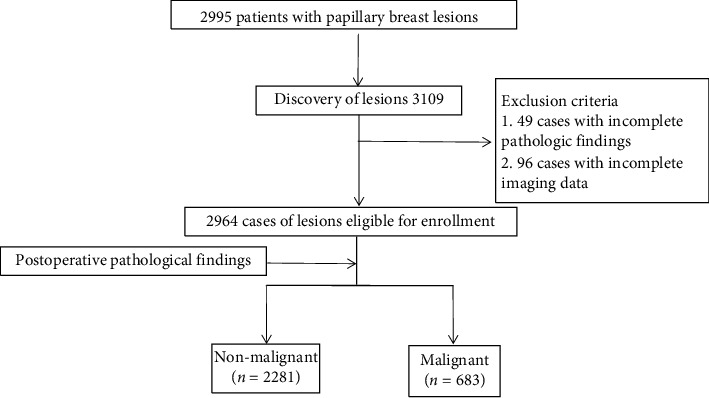
Flow diagram for the study.

The clinicopathologic data of 2995 patients who underwent open breast surgery at the Harbin Medical University Cancer Hospital between January 2010 and December 2016 and were diagnosed as “papillary lesions” in the postoperative pathology results were collected, respectively. Inclusion criteria are as follows: (1) pathologic diagnosis of PBLs and (2) imaging time (interval between performing surgery and the last preoperative imaging study, use of ultrasound and/or mammography) < 1 week. Exclusion criteria are as follows: (1) incomplete clinicopathological and imaging data and (2) concurrent presence of malignant tumors in other sites. 2964 cases were finally analyzed (Figure 1).

### 2.2. Cohort Definition and Data Collection

All patients underwent breast surgery and were re-evaluated by two experienced pathologists. The patients underwent surgery for the following reasons: imaging studies suggesting that the mass occurred as an intraductal occupancy; ultrasound suggesting a BI-RADS 4 classification (assessed through different aspects, such as shape, orientation, margin, echo pattern, and calcification) [11]; and strong surgical advocacy by the patients with mass exceeding 1 cm. According to the WHO histological classification of breast tumors [1], papillary lesions of the breast are classified into intraductal papilloma, papillary ductal carcinoma in situ, encapsulated papillary carcinoma, solid papillary carcinoma (in situ and invasive), and invasive papillary carcinoma. Patients were categorized into nonmalignant and malignant groups based on postoperative pathologic findings. The number of masses was determined based on ultrasound findings. Isolated lesions in the same ductal system were considered single, and ≥ two were considered multiple. For patients with recurrence after previous treatment admission, the recurred lesion was defined as the same if the mass appeared in the same ductal system in the ipsilateral breast; in all other cases (recurrence in the contralateral breast or bilateral concurrent disease), multiple masses were defined, and therefore, the number of patients enrolled was less than the number of masses. The locality of the lesion was determined based on the ultrasound findings. Lesions within 1 cm of the nipple were defined as central lesions, and any distant locations were defined as peripheral.

### 2.3. Statistical Analysis

The above statistical analyses were performed using IBM SPSS Version 27.0. The categorical variables were shown as frequencies and proportions and compared with the Chi-square tests. All factors with *p* < 0.05 in Chi-square tests were taken and further analyzed using multivariable logistic analysis to explore the independent risk factors affecting the malignant PBLs. *p* < 0.05 was considered a statistically significant difference.

## 3. Results

### 3.1. Clinicopathological Characteristics of the Patient

In this study, 2964 cases of PBLs were included, of which 2281 (77.0%) were in the nonmalignant group and 683 (23.0%) were in the malignant group. 42% (405/955) of patients aged ≥ 50 years were malignant, and 1101 patients showed palpable tumors, of which 453 (41.1%) were malignant PBLs. The rest of the clinicopathologic features are shown in Table 1.

### 3.2. Univariate and Multivariate Analyses

**Figure 2:**
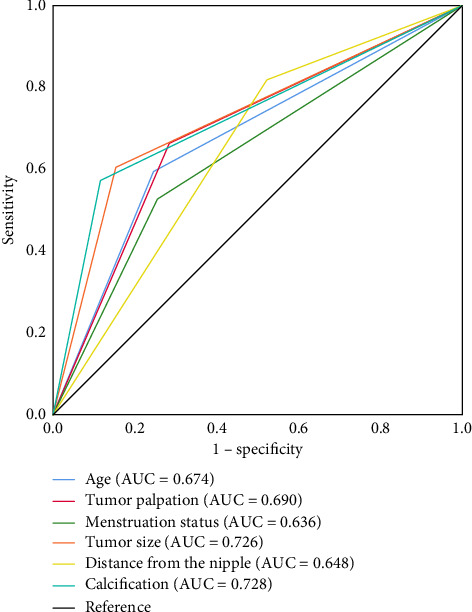
Assessment of the predictive power of six independent risk factors. AUC, area under the ROC curve.

The results of univariate analysis showed that age, tumor palpation, nipple discharge, menstruation status, tumor size, distance of the tumor from the nipple, and tumor with calcification may be associated with malignant PBLs (all *p* < 0.05). However, family history of malignant breast tumors, personal history of malignant breast tumors, and number of tumors were not significantly associated with malignant PBLs (all *p* > 0.05) (Table 1). As shown in Table 2, the factors that may be associated with malignant PBLs were included in the multivariate logistic analysis as age ≥ 50 years (OR = 2.724, 95% CI 2.131–3.483), palpable tumor (OR = 1.546, 95% CI 1.131–2.113), postmenopausal (OR = 1.829, 95% CI 1.425–2.349), tumor size ≥ 15 mm (OR = 3.884, 95% CI 2.839–5.313), peripheral lesions (OR = 2.904, 95% 2.241–3.764), and tumor with calcification (OR = 7.013, 95% CI 5.564–8.838) (all *p* < 0.05). We further validated the ability of the above six independent risk factors to predict malignant PBLs using receiver operating characteristic (ROC) curves. As shown in Figure 2, the area under the ROC curves (AUC) for age, tumor palpation, menstruation status, tumor size, distance from the nipple, and tumor with calcification were 0.674, 0.690, 0.636, 0.726, 0.648, and 0.726, respectively. These results further confirmed the predictive ability of these independent risk factors for malignancy of PBLs.

## 4. Discussion

In this study, we explored the independent risk factors affecting malignant PBLs by analyzing clinical variables in 2964 patients with PBLs. Finally, we selected six independent risk factors associated with malignant PBLs: ≥ 50 years, postmenopausal, palpable tumor, tumor size ≥ 15 mm, peripheral tumor, and tumor with calcification. The ROC curve verifies that the six factors could independently predict malignant PBLs (Figure 2). Besides, as shown in Table 3, there were 731 cases without the above risk factors, of which only 25 lesions were malignancies, with a malignancy probability of only 3.4%. For this low-risk group, perhaps patients would benefit more by choosing active surveillance instead of surgical treatment. In addition, patients with PBLs have a progressively higher risk of malignancy as risk factors accumulate. Thus, patients with one or more risk factors are more likely to benefit from surgical excision.

### 4.1. Clinical Factors

In previous studies, age was considered an independent risk factor for predicting malignant PBLs [12–14]. Similarly, in our study, it was found that the risk of malignancy in patients aged ≥ 50 years was 2.724 times higher than that in patients aged < 50 years (*p* < 0.001). As shown in Figure 2, the ROC curve confirms the age's independent discrimination and predictive capacity for malignant PBLs, with AUC values of 0.674.

Brennan et al. concluded that the probability of malignant PBLs was higher in postmenopausal women [15]. In this study, 74.5% of patients in the nonmalignant group were premenopausal (1699/2281), and 52.7% of patients in the malignant group were postmenopausal (360/683) corresponding to the former paper. Statistical analysis showed an 82.9% increase in the relative risk of malignancy of PBLs in postmenopausal women (OR = 1.829, 95% CI 1.425–2.34, *p* < 0.001).

In our study, 59.1% of patients had palpable tumors. The analysis showed that the risk of malignant PBLs with palpable was 1.546 times higher than that of nonpalpable tumors (OR = 1.546, 95% CI 1.131–2.113, *p*=0.006). In Li et al.'s study, results showed that 748 of 2290 patients with PBLs with palpable tumors developed cancer. In comparison, only 69 of 2160 patients with nonpalpable tumors developed cancer, with the difference reaching statistical significance (*p* < 0.01) [16]. Similarly, a study of 250 cases from Korea also demonstrated that palpable tumors are independent risk factors for malignant PBLs [11].

Whether nipple discharge predicts malignant PBLs is not uniformly agreed upon by researchers. Most scholars have shown that nipple discharge does not increase the risk of malignancy in PBLs [14, 14, 17], which is supported by our study (*p*=0.599). According to NCCN guideline v2.2024, abnormal nipple discharge is defined as persistent, spontaneous uniductal, unilateral bloody, or clear nipple discharge. Although family history and personal history are associated with breast cancer, we did not find a clear association between family history and personal history and malignant PBLs in our study. This is similar to the findings of some other studies [15, 17, 18]. However, Abbassi-Rahbar et al. reported that 28.6% of malignant cases had a previous diagnosis of ipsilateral breast cancer, and further studies confirmed that the risk of escalation was significantly higher when PBLs were located in the same quadrant as the ipsilateral malignancy (*p*=0.023) [3]. The difference in our findings may be because the present study only explored the effect of personal history on the malignancy of PBLs and did not breakdown the association of history of ipsilateral and contralateral breast cancer with malignant PBLs, respectively.

### 4.2. Imaging Factors

Glenn et al. reported that the probability of malignancy in patients with PBLs with a mass < 15 mm was only 4.7% [19] and the size of the mass was determined by the ultrasound data, taking the longest diameter. Similarly, Kil et al. found that most PBLs were ≥ 15 mm, while most benign PBLs were < 15 mm [20]. Our study confirmed that when the tumor size was ≥ 15 mm, the risk of malignancy was 3.903 times higher than that of patients with tumors < 15 mm (*p* < 0.0001). This result is in line with several other studies confirming that the risk of malignant PBLs increases with the tumor size.

PBLs are categorized into central and peripheral lesions, and previous studies have elucidated that peripheral PBLs have a higher probability of malignancy than the central type [14]. Several studies have also explored the effect of the tumor's location on the malignancy of PBLs and found that the tumor's location in the malignant group was further away from the nipple [2, 21]. In our study, there were 1213 patients in the central type, with 124 (10.2%) malignancy and 1751 patients in the peripheral type, of which 559 (31.9%) malignancy. The difference was statistically significant (OR = 2.951, 95% CI 2.274–3.829, *p* < 0.0001).

A study by Oyama and Koerner showed that multiple PBLs would have a higher risk of malignancy [22], in contrast to Gutman et al., who concluded that the number of PBLs does not affect its risk of malignancy [23]. Similarly, our study found that the number of tumors does not affect malignant PBLs.

We explored the effect of tumors accompanied by calcification on malignant PBLs. Of the 2964 cases enrolled, 655 lesions were accompanied by calcification, of which 391 (59.7%) were proved to be malignant. Only 292 (12.6%) of 2309 cases in the group without calcification were malignant. Statistical analysis showed that the risk of malignancy in the calcified group was 7.021 times higher than that in the noncalcified group (OR = 7.021, 95% CI 5.568–8.855, *p* < 0.0001). The same results were confirmed in other studies [24].

This study is a single-center retrospective analysis, but our study is the most extensive sample study on PBLs to date, and six independent risk factors were screened to predict malignant PBLs. Besides, we still need prospective experiments to validate our findings.

## 5. Conclusion

This study examined the clinical variables of malignant and nonmalignant PBLs in patients. The findings revealed that older patients (aged 50 years and above), postmenopausal, with a palpable tumor, a tumor size of 15 mm or greater, a peripheral tumor, and a tumor with calcification were identified as independent risk factors associated with malignant PBLs. The aforementioned findings support the implementation of early surgical excision in patients exhibiting the identified risk factors. Conversely, patients presenting with a minimal number of risk factors (0–3) may benefit from regular imaging surveillance. Furthermore, the probability of malignant PBLs in the absence of the aforementioned risk factors was found to be only 3.4%. This substantiates the reliability of the present study.

## Figures and Tables

**Table 1:** Clinicopathologic imaging characteristics and univariate analysis of 2964 patients with papillary breast lesions.

Clinicopathologic imaging characteristics	Nonmalignant (*n* = 2281)	Malignant (*n* = 683)	All (*n* = 2964)	*X* ^2^	*p*
Age (years)				291.291	< 0.0001
< 50	1721 (86.1%)	277 (13.9%)	1998		
≥ 50	560 (58.0%)	406 (42.0%)	966		
Tumor palpation				323.653	< 0.0001
Unpalpable	1633 (87.7%)	230 (12.3%)	1863		
Palpable	648 (58.9%)	453 (41.1%)	1101		
Nipple discharge				47.15	< 0.0001
Absent	1516 (73.4%)	548 (26.6%)	2064		
Present	765 (85.0%)	135 (15.0%)	900		
Family history				0.887	0.346
Absent	2065 (77.2%)	610 (22.8%)	2675		
Present	216 (74.7%)	73 (25.3%)	289		
Personal history				0.263	0.608
Absent	2142 (76.9%)	645 (23.1%)	2787		
Present	139 (78.5%)	38 (21.5%)	177		
Menstruation status				179.276	< 0.0001
Premenopausal	1699 (84.0%)	323 (16.0%)	2022		
Postmenopausal	582 (61.8%)	360 (38.2%)	942		
Tumor size (mm)				559.889	< 0.0001
< 15	1931 (87.7%)	270 (12.3%)	2201		
≥ 15	350 (45.9%)	413 (54.1%)	763		
Number of tumor				1.32	0.251
Solitary	1810 (77.4%)	528 (22.6%)	2338		
Multiple	471 (75.2%)	155 (24.8%)	626		
Distance from the nipple (cm)				190.318	< 0.0001
Central lesions	1089 (89.8%)	124 (10.2%)	1213		
Peripheral lesions	1192 (68.1%)	559 (31.9%)	1751		
Calcification				636.936	< 0.0001
Absent	2017 (87.4%)	292 (12.6%)	2309		
Present	264 (40.3%)	391 (59.0%)	655		

**Table 2:** Multivariate logistic regression analysis of papillary breast lesions based on clinical impact characteristics.

Clinical imaging characteristics	OR	95% CI	*p*
Age (years)	2.724	2.131–3.483	< 0.0001
< 50			
≥ 50			
Tumor palpation	1.546	1.131–2.113	0.006
Unpalpable			
Palpable			
Nipple discharge	1.075	0.8218–1.114	0.599
Absent			
Present			
Menstruation status	1.829	1.425–2.349	< 0.0001
Premenopausal			
Postmenopausal			
Tumor size (mm)	3.884	2.839–5.313	< 0.0001
< 15			
≥ 15			
Distance from the nipple (cm)	2.904	2.241–3.764	< 0.0001
Central lesions			
Peripheral lesions			
Calcification	7.013	5.564–8.838	< 0.0001
Absent			
Present			

**Table 3:** Probability of malignancy in patients with 0–6 risk factors for papillary breast lesion.

Cumulative risk factors	Probability of malignancy
0	3.4% (25/731)
1	8.2% (65/787)
2	19.6% (128/653)
3	41.3% (166/401)
4	68.8% (150/218)
5	84.9% (135/159)
6	93.3% (14/15)

## Data Availability

The data that support the findings of this study are available from the corresponding author upon reasonable request.

## Nomenclature

PBLs: Papillary breast lesions
ROC: Receiver operating characteristic
OR: Odds ratio
CI: Confidence interval
OS: Overall survival
